# Prevalence and Correlates of Firearm Screening and Counseling in Primary Care in Southeast Michigan

**DOI:** 10.1016/j.focus.2025.100370

**Published:** 2025-05-23

**Authors:** Joseph B. Ladines-Lim, Magdalena Hecht, Caleb Arthur, Autumn Pu, Melissa Ross, Kayla Secrest, Aaron Sifuentes, Justin Litzner, Jennifer Stojan, Michelle Degli Esposti, Jennifer Meddings

**Affiliations:** 1 Division of Infectious Diseases, Department of Medicine, Penn Medicine, University of Pennsylvania, Philadelphia, Pennsylvania; 2 Department of Family Medicine, Michigan Medicine, University of Michigan, Ann Arbor, Michigan; 3 Kaiser Permanente, Pinole, California; 4 Department of Internal Medicine, Michigan Medicine, University of Michigan, Ann Arbor, Michigan; 5 Division of Pulmonary and Critical Care, Department of Medicine, Feinberg School of Medicine, Northwestern Medicine, Chicago, Illinois; 6 Division of Cardiology, Department of Internal Medicine, Michigan Medicine, University of Michigan, Ann Arbor, Michigan; 7 Division of Cardiology, Department of Internal Medicine, University of Iowa, Iowa City, Iowa; 8 Michigan Medicine, University of Michigan, Ann Arbor, Michigan; 9 Department of Pediatrics, Michigan Medicine, University of Michigan, Ann Arbor, Michigan; 10 Institute for Firearm Injury Prevention, University of Michigan, Ann Arbor, Michigan; 11 Center for Clinical Management Research, Veterans’ Affairs Ann Arbor Healthcare System, Ann Arbor, Michigan

**Keywords:** Firearm safety, screening, counseling, primary care, health maintenance examination

## Abstract

**Introduction:**

The American College of Physicians recommends firearm screening and safety counseling for high-risk patients, although frequency of practice is unclear. The authors evaluated the prevalence and correlates of firearm screening and counseling during health maintenance exams.

**Methods:**

The authors performed a cross-sectional analysis of all health maintenance exams for patients aged ≥18 years at an academic center in Southeast Michigan from September 2021 to February 2022. Primary outcomes included documentation of attempted firearm screening and counseling after positive screens. Multinomial logistic regression models estimated the associations between primary outcomes and patient and clinician characteristics using multilevel modeling to account for interclinic variability.

**Results:**

In 27,686 health maintenance exams across 17 clinic sites, attempted screening was documented in 99.8% of visits, principally through routine questionnaires, with positive, negative, or no responses in 15.2%, 48.0%, and 36.8%, respectively. One outlier clinic site documented attempted screening in only 0.9% of health maintenance exams and was excluded. Counseling occurred in 1.1% of positive screens; clinic sites accounted for 36.3% of variability. Counseling was less likely for females (AOR=0.65; 95% CI=0.51, 0.82), patients aged ≥65 years (AOR=0.31; 95% CI=0.21, 0.47), and Asian patients (AOR=0.31; 95% CI=0.16, 0.61) and more likely for internal medicine–pediatrics/family medicine clinicians than internal medicine clinicians (AOR=4.42; 95% CI=1.99, 9.82).

**Conclusions:**

Clinicians documented attempted firearm screening in nearly all health maintenance exams but seldom counseling, which varied across sites and was linked to patient sociodemographic characteristics and clinician specialty as opposed to high-risk clinical features such as psychiatric disorder. Findings suggest that training and quality improvement efforts are needed to ensure appropriate risk-based counseling.

## Highlights

• At an academic medical center in Southeast Michigan, firearm screening was common.
• Routine questionnaires accounted for nearly all screening at health maintenance exams.
• Safety counseling was rare and associated with sociodemographic characteristics.
• Counseling was not associated with high-risk features, for example, psychiatric disorder.
• Quality improvement efforts are warranted to ensure risk-based counseling.

## INTRODUCTION

A public health approach is needed to address the ongoing firearm violence epidemic in the U.S.,^1^ including increasingly engaging clinicians in firearm injury prevention through screening and appropriate safety counseling.^2^, ^3^, ^4^ Following evidence of the effectiveness of firearm counseling in reducing firearm-related injuries,^5^ the American College of Physicians (ACP) recommends that clinicians advise on the risk of firearms, especially to those with high-risk features, such as psychiatric and substance use disorder.^6^ However, many clinicians feel that discussing firearms falls outside their responsibilities,^2^^,^^7^ and it is unclear whether they do this in practice.^7^^,^^8^

At the authors’ institution, a large suburban tertiary academic medical center in Southeast Michigan, firearm screening occurs during health maintenance exams (HMEs) through various methodologies, including routine questionnaires provided by ancillary staff in person during the visit or electronically before the visit or by the clinicians themselves during the visit. The authors aimed to characterize the prevalence of firearm screening and counseling during HMEs at the authors’ institution through retrospective chart review and determine whether clinicians had a higher prevalence of screening and counseling for patients with high-risk features, such as known psychiatric disorder or substance use disorder, in keeping with guidelines stated in the literature and by the ACP.^6^^,^^8^

## METHODS

### Study Sample

The authors abstracted data from the electronic medical record at the authors’ institution for all new patient and return visit HMEs for patients aged ≥18 years seen by a clinician in internal medicine, family medicine, or internal medicine–pediatrics across 18 individual clinics from September 1, 2021 to February 28, 2022. Data included patient characteristics (age, sex, race/ethnicity, diagnosis codes from active problem list), clinician specialty and training level (advanced practice provider, resident or fellow physician, or attending physician), and any documented screening and counseling through patient responses to routine questionnaires and/or review of progress notes (Appendix, available online). Using diagnosis codes for each patient, the authors identified any history of psychiatric disorder, substance use, dementia or intellectual disability, self-harm, suicide attempt, suicidal or homicidal ideation, and comorbidities for calculation of the Charlson Comorbidity Index (Appendix, available online).^9^ The IRB of the University of Michigan Medical School approved this study with the understanding that data were deidentified and then destroyed after study completion (Study Number HUM00220241). The authors followed STROBE guidelines for cross-sectional studies.^10^

### Measures

Primary outcomes included (1) any attempted firearm screening and (2) safety counseling associated with positive screening in the chart. Secondary outcomes included patient responses to firearm screening (*yes, no*, and screened and did not respond) and counseling associated with negative screening, lack of screening response despite documented attempted screening, or no screening performed at all (Appendix Table 1, available online). The authors defined counseling as any text in the progress note stating that counseling was performed or alluding to firearm storage practices (e.g., locks, safes, firearms out of the home), regardless of extent of detail, which could be free form text or from a prepopulated template (Appendix, available online).

### Statistical Analysis

The authors used multinomial logistic regression models, accounting for multicollinearity among covariates, to estimate associations of primary outcomes with patient and clinician information described earlier, using multilevel modeling to account for patients nested within different clinic sites. The authors employed 2-sided hypothesis tests with α=0.05 and conducted analyses using RStudio, Version 4.3.0 (R Foundation for Statistical Computing, Vienna, Austria).

## RESULTS

The authors initially reviewed 30,772 HME visits (Appendix Table 1, available online). Upon review of screening by clinic site, the authors found that 1 site performed screening in 0.9% of visits, whereas the other 17 ranged from 99.1% to 100% (Appendix Table 2, available online). The authors thus excluded this clinic site (clinic #1 in Appendix Table 2, available online) as an outlier from the overall analysis, leaving 27,686 available for final analysis (Table 1 presents the sample characteristics**)**. Attempted firearm screening was documented in 99.8% of visits, with positive, negative, or no responses in 15.2%, 48.0%, and 36.8% of screens, respectively (Table 2). Of note, routine questionnaires administered by ancillary staff or submitted electronically before the visit accounted for all screens but 1 (i.e., routine questionnaires were administered in 27,635 of 27,636 visits with screening); still, clinicians documented some attempted screening in a small minority of these visits (2,117 of 27,363 visits or 7.7%).^7^ Given the near universality of screening with the exception of 1 outlier clinic site, the authors deferred on performing a multinomial logistic regression model for this outcome. Among those screened, the authors did note differences in sociodemographic characteristics between those who responded to screens (either positively or negatively) and those who did not respond to screens, with the latter being older, comprised more male participants, and comprised more non-Hispanic Whites and fewer minority groups except non-Hispanic Asians (Appendix Table 3, available online).

**Table 1:** Patient Sociodemographic and Clinician Characteristics of Cohort, Excluding 1 Outlier Clinic Site of 18 Total

Characteristics	*n* (%)		
	Overall (N=27,686)^a^	Internal medicine (*n*=14,241)	Family medicine and internal medicine–pediatrics (*n*=13,445)
Patient sociodemographic characteristics			
Female sex	15,672 (50.9%)	7,780 (44.9%)	7,892 (58.7%)
Age group, years			
18–44	8,409 (27.3%)	3,571 (20.6%)	4,838 (36.0%)
45–64	10,657 (34.6%)	5,422 (31.3%)	5,235 (38.9%)
≥65	8,620 (28.0%)	5,248 (30.3%)	3,372 (25.1%)
Race and ethnicity			
Hispanic, any race	877 (2.8%)	355 (2.0%)	522 (3.9%)
Non-Hispanic, Asian	2,191 (7.1%)	982 (5.7%)	1,209 (9.0%)
Non-Hispanic, Black	2,082 (6.8%)	764 (4.4%)	1,318 (9.8%)
Non-Hispanic, other	889 (2.9%)	433 (2.5%)	456 (3.4%)
Non-Hispanic, White	21,647 (70.3%)	11,707 (67.6%)	9,940 (73.9%)
Patient clinical characteristics			
History of psychiatric disorder, suicide attempt, or suicidal or homicidal ideation	9,108 (29.6%)	4,270 (24.6%)	4,838 (36.0%)
History of dementia	535 (1.7%)	277 (1.6%)	258 (1.9%)
History of substance use disorder	2,665 (8.7%)	1,034 (6.0%)	1,631 (12.1%)
Charlson Comorbidity Index score			
<2	21,163 (68.8%)	10,452 (60.3%)	10,711 (79.7%)
≥2	6,523 (21.2%)	3,789 (21.9%)	2,734 (20.3%)
Clinician characteristics			
Clinician level			
Advanced practice provider	2,700 (8.8%)	1,105 (6.4%)	1,595 (11.9%)
Attending physician	20,875 (67.8%)	11,446 (66.1%)	9,429 (70.1%)
Resident or fellow physician	4,111 (13.4%)	1,690 (9.8%)	2,421 (18.0%)
Clinician specialty			
Internal Medicine	14,241 (51.4%)	14,241 (100%)	0 (0%)
Family Medicine and Internal Medicine–Pediatrics	13,445 (48.6%)	0 (0%)	13,445 (100%)

a This sample excludes 1 of 18 clinic sites given outlier result of near zero screening as shown in Appendix Table 1 (available online).

**Table 2:** Prevalence of Firearm Screening, Screening Responses, and Firearm Safety Counseling by Specialty

Outcome	*n* (%)		
	Overall (N=27,686)^a^	Internal medicine (*n*=14,241)	Family medicine and internal medicine–pediatrics (*n*=13,445)
Any screening	27,636 (99.8%)	14,236 (100%)	13,400 (99.7%)
Positive screen^b^	4,191 (15.2%)	2,422 (17.0%)	1,769 (13.2%)
Negative screen^b^	13,275 (48.0%)	7,225 (50.8%)	6,050 (45.1%)
No response^b^	10,170 (36.8%)	4,589 (2.2%)	5,581 (41.6%)
Counseling	912 (3.3%)	142 (1.0%)	770 (5.7%)
Any screening^c^	912 (100%)	142 (100%)	770 (100%)
Positive screening^d^	293 (32.1%)	50 (35.2%)	243 (31.6%)
Negative screening^d^	292 (32.0%)	49 (34.5%)	243 (31.6%)
No response to screening^d^	327 (35.9%)	43 (30.3%)	284 (36.9%)

a This sample excludes 1 of 18 clinic sites given outlier result of near zero screening as shown in Appendix Table 1 (available online).

b Denominator of screening responses is total number of screens rather than the entire sample.

c Denominator of counseling with any screening is total number of any counseling.

d Denominator of counseling with positive screening, negative screening, or no response to screening is total number of counseling with any screening.

**Figure 1:**
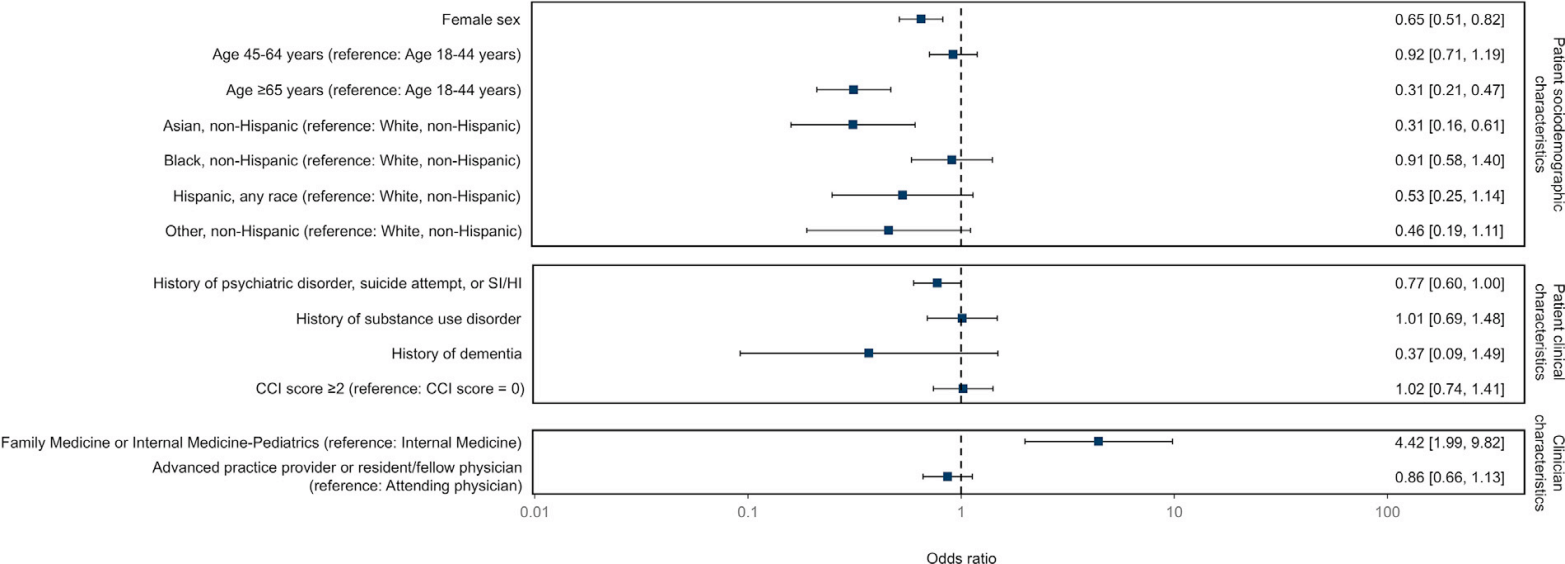
ORs of logistic regression model correlates of firearm safety counseling given positive screening, excluding 1 outlier clinic site of 18 total.

*Note:* ORs are adjusted for all covariates in the model.

In contrast, clinicians documented counseling in 1.1% of visits with positive screens (293 of 27,636) (details on statistics are presented for Counseling, subcategory Positive screening and Any screening in Table 2), with variability across individual clinics accounting for 36.3% of outcome. Of note, although the authors focused on counseling associated with positive screening, they found a significant proportion of counseling (67.9%) to be associated with either negative screening or no response to screening (Table 2). Regression models showed that counseling with positive screening was less likely for female patients (AOR=0.65; 95% CI=0.51, 0.82), patients aged ≥65 years (AOR=0.31; 95% CI=0.21, 0.47), and non-Hispanic Asian patients (AOR=0.31; 95% CI=0.16, 0.61) and more likely for Internal Medicine–Pediatrics and Family Medicine clinicians versus Internal Medicine clinicians (AOR=4.42; 95% CI=1.99, 9.82) (Figure 1). In addition, counseling was less likely for patients with psychiatric disorder or history of suicide attempt or suicidal or homicidal ideation, although this did not meet criteria for statistical significance (AOR=0.77; 95% CI=0.60, 1.00, *p*=0.052). The authors also conducted a sensitivity analysis, including the outlier clinic that had been excluded owing to its aberrantly low prevalence of screening (Appendix Table 2, available online) and found that ORs were very similar when including this outlier clinic (Appendix Figure 1, available online).

## DISCUSSION

In this cross-sectional analysis of HMEs at a large suburban tertiary academic medical center in Southeast Michigan (excluding 1 outlier site of 18 total), documentation of attempted screening was near universal (99.8%), largely owing to routine questionnaires, whereas counseling was rare (1.1%). The fact that there was documented in-visit screening in 7.7% of visits, even with routine questionnaires being administered in nearly all visits with screening, suggests that either clinicians elected to perform their own screening or they were not aware of routine questionnaire results, which would be plausible given prior study results at this same academic medical institution showing that this was true for nearly a third of clinicians surveyed at a subset of the clinics included in this study.^7^ The authors’ prior work also highlighted numerous barriers to addressing firearms with patients in the clinic, including time constraints, lack of related training, and discomfort for fear of jeopardizing the patient/provider relationship, which is consistent with other past literature^7^^,^^11^, ^12^, ^13^; some clinicians also noted not always documenting these conversations even if they did provide counseling on the topic, which could also explain the low prevalence of documented counseling in this study.^7^ Conversely, the authors also note that when counseling was documented, it was often done so without a positive screen, either with a negative screen or no response to screening, whereas it is plausible that clinicians may have done this as a pre-emptive measure in certain clinical situations (e.g., patient deemed to be higher risk for self-harm or harm to others even if not known to have firearm access); it is also possible that such documented counseling may have been erroneous and/or taken from a template and not deleted if the conversation was not had.

It is worth noting that the excluded clinic is the exception in not having implemented routine questionnaires to this end and similarly had very little counseling documented (Appendix Table 2, available online), which perhaps explains why including this clinic had little impact on the multivariable analysis (Appendix Figure 1, available online); whether routine questionnaires are administered is at the discretion of individual clinics, as opposed to mandated by the health system. It is also worth noting the sociodemographic differences between those who responded to screening and those who did not (Appendix Table 3, available online); although this was not the focus of this work, this could certainly be a fruitful path for future study.

Despite ACP recommendations, high-risk features (e.g., psychiatric disorder, substance use disorder, dementia) were not associated with increased documented screening or safety counseling; rather, such history was associated with decreased counseling with near statistical significance. Certain sociodemographic characteristics (i.e., age, sex, race/ethnicity) and clinician specialty (categorical internists) were also associated with decreased counseling. Reasons for discrepancies in practice are unknown and possibly myriad (e.g., implicit bias, specialty-specific norms, variable template use for documentation); the barriers to addressing the topic in clinic mentioned earlier, including time constraints, lack of related training, and topic discomfort, further confound study results and could be contributory. Determining more precisely which factors result in discrepancies in screening and counseling and seemingly lack of increased attention to high-risk patients certainly warrants future study, both through quantitative and qualitative approaches. The study findings add to those of other recent studies on firearm access screening and safety counseling for adult patients,^7^^,^^11^, ^12^, ^13^ suggesting the need for clinician education and quality improvement efforts to ensure uniform provision of screening and counseling across sociodemographic groups and clinics with appropriate adjustments for high-risk patients and documentation thereof.

The authors note briefly that the positive response rate of 15.2% is much less than a previously estimated rate of 40.2% in the State of Michigan in a 2020 study.^14^ Although it is possible that the reported positive response rate is due to underestimation, it is also possible that the authors’ institution’s patient population differs from the overall statewide population in this regard, given socioeconomic and possibly cultural differences between Southeast Michigan and the remainder of the state.

### Limitations

This study has several limitations, including the use of data from a single academic medical center, limiting generalizability; not adjusting for other potential confounders (e.g., income level); and reliance on diagnosis codes in the medical chart, which may not accurately reflect actual patient medical history. The authors also limited their scope to HME visits, not including separate return visits during which clinicians could plausibly focus more on high-risk features, such as major depressive disorder, and appropriately perform lethal means screening and safety counseling. Finally, there may be discordance between documentation and actual practice; it is plausible that clinicians may be providing counseling but not documenting this practice and vice versa.

## CONCLUSIONS

In summary, this study at a large suburban tertiary academic medical center in Southeast Michigan demonstrated near universal attempted firearm screening during HMEs with 1 notable clinic site in exception, suggesting systemic factors at the clinic level that could be addressed through well-implemented processes such as routine questionnaires. Safety counseling was rare and correlated with certain patient sociodemographic characteristics and clinician specialty. Future training and quality improvement efforts should focus on ensuring that clinic sites and individual clinicians provide consistent screening and counseling with particular attention to high-risk groups in keeping with public health recommendations.^6^^,^^8^

## Appendix Supplementary materials

Image, application 1

Image, application 2

Image, application 3

Image, application 4

Image, application 5

Image, application 6

Image, application 7

Image, application 8

Image, application 9

Image, application 10

Image, application 11

Image, application 12

Image, application 13

Image, application 14

Image, application 15

Image, application 16

Image, application 17

Image, application 18

Image, application 19

Image, application 20

Image, application 21

Image, application 22

Image, application 23

## Footnotes

Supplementary material associated with this article can be found in the online version at doi:10.1016/j.focus.2025.100370.
