# Supplementary material for: Prevalence and Correlates of Firearm Screening and Counseling in Primary Care in Southeast Michigan
Source: AJPM Focus. 2025 May 23;4(6):100370. doi: 10.1016/j.focus.2025.100370 (PMC12480869; doi:10.1016/j.focus.2025.100370)
Supplement: Supplementary file 1 [file mmc1.docx]

**APPENDIX**

We extracted data from our electronic medical record using the Epic Clarity database. We describe the variables used in our analysis below.

Patient sociodemographic and clinical characteristics:

1. Age in years
2. Biologic sex: female or male.
3. Race/ethnicity: Hispanic, any race; non-Hispanic Asian; non-Hispanic Black; non-Hispanic Other; non-Hispanic White.
4. Active medical problems based on those listed in problem list at time of encounter. We used ICD-10 diagnosis codes to map each problem to one of the diagnosis categories in the Pediatric Comorbidity Index (PCI).^1^ These codes are listed in the following Excel files which are also attached separately:
   1. ICD-10-CM_medicine_AIDS.xlsx
   2. ICD-10-CM_medicine_any_malignancy.xlsx
   3. ICD-10-CM_medicine_CHF.xlsx
   4. ICD-10-CM_medicine_CVD.xlsx
   5. ICD-10-CM_medicine_dementia.xlsx
   6. ICD-10-CM_medicine_diabetes.xlsx
   7. ICD-10-CM_medicine_diabetes_complications.xlsx
   8. ICD-10-CM_medicine_hemiplegia_quadriplegia.xlsx
   9. ICD-10-CM_medicine_HIV.xlsx
   10. ICD-10-CM_medicine_liver_disease_mild.xlsx
   11. ICD-10-CM_medicine_liver_disease_moderate_severe.xlsx
   12. ICD-10-CM_medicine_metastatic_solid_tumor.xlsx
   13. ICD-10-CM_medicine_myocardial_infarction.xlsx
   14. ICD-10-CM_medicine_PUD.xlsx
   15. ICD-10-CM_medicine_pulmonary_disease.xlsx
   16. ICD-10-CM_medicine_PVD.xlsx
   17. ICD-10-CM_medicine_renal_disease.xlsx
   18. ICD-10-CM_medicine_renal_disease_severe.xlsx
   19. ICD-10-CM_medicine_rheumatic_disease.xlsx

Of note, we also mapped codes to the following categories: psychiatric disorder, substance use, and history of self-harm or suicidal ideation or homicidal ideation. These shared some but not complete overlap with some of the categories used for the PCI. The Excel files used for this purpose are listed below and also attached separately:

1. ICD-10-CM_medicine_psychiatric.xlsx
2. ICD-10-CM_medicine_substance_use.xlsx
3. ICD-10-CM_medicine_SHSIHI.xlsx

Clinician characteristics:

1. Training level: attending physician, resident or fellow physician, and advanced practice provider.
2. Specialty: Categorical Internal Medicine, Family Medicine, or combined Internal Medicine-Pediatrics.

Of note, we used EMERSE to search through documentation to identify outcomes. We used this as an adjunctive method, not the sole method for identifying outcomes. We used the following terms for WCEs for patients included in the cohort: firearm*, gun*, weapon*.

*Screening:*

When examining documented screening, we looked at both free form text and routine questionnaires. Below are all possible questions on any routine questionnaires that may have been administered to this end.

- “Are there any guns in the home?”
- “Does your family keep guns in the home?”
- “Do you have a gun at home?”

*Counseling:*

When examining documented counseling related to firearm safety, we counted any text in the progress note that alluded to this, regardless of whether from free text or an already available template and regardless the level of detail included. Below are a few select examples.

- *Example 1 (template):* “Injury prevention: Discussed safety belts, safety helmets, smoke detector, smoking near bedding or upholstery and firearm safety (gun safe, trigger locks, ammunition stored separate from weapon”
- *Example 2 (free text):* “Gun stored safely”
- *Example 3 (free text):* “Gun at home stored with a lock, unloaded, and in a safe with ammunition stored separately”

**Table 1.** Prevalence of firearm screening, screening responses, and firearm safety counseling by specialty, including all 18 clinics.

| **Outcome** | **No. (%)** | | |
| --- | --- | --- | --- |
|  | Overall  (N=30,772) | Internal Medicine  (N=17,327) | Family Medicine and Internal Medicine-Pediatrics  (N=13,445) |
| **Any screening** | 27,664 (89.9%) | 14,264 (82.3%) | 13,400 (99.7%) |
| Positive screen^a^ | 4,199 (15.2%) | 2,430 (17%) | 1,769 (13.2%) |
| Negative screen^a^ | 13,293 (48.1%) | 7,243 (50.8%) | 6,050 (45.1%) |
| No response^a^ | 10,172 (36.8%) | 4,591 (32.2%) | 5,581 (41.6%) |
| **Counseling** | 921 (3.0%) | 151 (0.9%) | 770 (5.7%) |
| Any screening^b^ | 917 (99.6%) | 147 (97.4%) | 770 (100.0%) |
| Positive screening^c^ | 297 (32.2%) | 54 (35.8%) | 243 (31.6%) |
| Negative screening^c^ | 293 (31.8%) | 50 (33.1%) | 243 (31.6%) |
| No response to screening^c^ | 327 (35.5%) | 43 (28.5%) | 284 (36.9%) |
| No screening^b^ | 4 (0.4%) | 4 (2.6%) | 0 (0.0%) |

^a^ Denominator of screening responses is total number of screens, rather than the entire sample.

^b^ Denominator of counseling with any screening or counseling with no screening is total number of any counseling.

^c^ Denominator of counseling with positive screening, negative screening, or no response to screening is total number of counseling with any screening.

**Table 2**. Prevalence of firearm screening, screening responses, and firearm safety counseling by specialty, divided by clinic site.

| **Outcome** | **No. (%)** | | | | | | | | | | | | | | | | | | |
| --- | --- | --- | --- | --- | --- | --- | --- | --- | --- | --- | --- | --- | --- | --- | --- | --- | --- | --- | --- |
|  | Overall  (N=30,772) | Clinic #1  (N=3,086) | Clinic #2  (N=1,505) | Clinic #3  (N=1,016) | Clinic #4  (N=2,902) | Clinic #5  (N=999) | Clinic #6  (N=1,463) | Clinic #7  (N=2,795) | Clinic #8  (N=2,328) | Clinic #9  (N=2,146) | Clinic #10  (N=1,381) | Clinic #11  (N=2,108) | Clinic #12  (N=1,443) | Clinic #13  (N=1,768) | Clinic #14  (N=1,403) | Clinic #15  (N=1,367) | Clinic #16  (N=847) | Clinic #17  (N=1,772) | Clinic #18  (N=443) |
| **Any screening** | 27,664  (89.9%) | 28  (0.9%) | 1,493  (99.2%) | 1,015  (99.9%) | 2,902  (100%) | 999  (100%) | 1,463  (100%) | 2,795  (100%) | 2,307  (99.1%) | 2,144  (99.9%) | 1,380  (99.9%) | 2,108  (100%) | 1,441  (99.9%) | 1,768  (100%) | 1,403  (100%) | 1,365  (99.9%) | 845  (99.8%) | 1,765  (99.6%) | 443  (100%) |
| Positive screen | 4,199  (15.2%) | < 10 | 290  (19.4%) | 123  (12.1%) | 897  (30.9%) | 99  (9.9%) | 114  (7.8%) | 341  (12.2%) | 238  (10.3%) | 303  (14.1%) | 273  (19.8%) | 346  (16.4%) | 207  (14.4%) | 292  (16.5%) | 150  (10.7%) | 169  (12.4%) | 67  (7.9%) | 214  (12.1%) | 68  (15.3%) |
| Negative screen | 13,293  (48.1%) | 18  (64.3%) | 583  (39%) | 643  (63.3%) | 1,566  (54%) | 500  (50.1%) | 527  (36%) | 1,302  (46.6%) | 1,149  (49.8%) | 992  (46.3%) | 707  (51.2%) | 591  (28%) | 844  (58.6%) | 1,073  (60.7%) | 712  (50.7%) | 586  (42.9%) | 373  (44.1%) | 836  (47.4%) | 291  (65.7%) |
| No response | 10,172  (36.8%) | < 10 | 620  (41.5%) | 249  (24.5%) | 439  (15.1%) | 400  (40%) | 822  (56.2%) | 1,152  (41.2%) | 920  (39.9%) | 849  (39.6%) | 400  (29%) | 1,171  (55.6%) | 390  (27.1%) | 403  (22.8%) | 541  (38.6%) | 610  (44.7%) | 405  (47.9%) | 715  (40.5%) | 84  (19%) |
| **Counseling** | 921  (3.0%) | < 10 | 124  (8.2%) | 37  (3.6%) | < 10 | < 10 | 132  (9%) | < 10 | 62  (2.7%) | 69  (3.2%) | 14  (1%) | 205  (9.7%) | 13  (0.9%) | 29  (1.6%) | 11  (0.8%) | 164  (12%) | < 10 | 27  (1.5%) | < 10 |
| Any screening | 917  (99.6%) | < 10 | 124  (100%) | 37  (100%) | < 10 | < 10 | 132  (100%) | < 10 | 62  (100%) | 69  (100%) | 14  (100%) | 205  (100%) | 13  (100%) | 29  (100%) | 11  (100%) | 164  (100%) | < 10 | 27  (100%) | < 10 |
| Positive screening | 297  (32.2%) | < 10 | 68  (54.8%) | 37  (100%) | < 10 | < 10 | 36  (27.3%) | < 10 | 10  (16.1%) | 13  (18.8%) | < 10 | 43  (21%) | > 10 | < 10 | 11  (100%) | 23  (14%) | < 10 | > 10 | < 10 |
| Negative screening | 293  (31.8%) | < 10 | 30  (24.2%) | 0  (0%) | < 10 | < 10 | 28  (21.2%) | 0  (0%) | 35  (56.5%) | 23  (33.3%) | < 10 | 42  (20.5%) | < 10 | > 10 | 0  (0%) | 90  (54.9%) | < 10 | > 10 | < 10 |
| No response to screening | 327  (35.5%) | 0  (0%) | 26  (21%) | 0  (0%) | 0  (0%) | < 10 | 68  (51.5%) | < 10 | 17  (27.4%) | 33  (47.8%) | < 10 | 120  (58.5%) | 0  (0%) | < 10 | 0  (0%) | 51  (31.1%) | < 10 | < 10 | < 10 |
| No screening | 4  (0.4%) | 4  (44.4%) | 0  (0%) | 0  (0%) | 0  (0%) | 0  (0%) | 0  (0%) | 0  (0%) | 0  (0%) | 0  (0%) | 0  (0%) | 0  (0%) | 0  (0%) | 0  (0%) | 0  (0%) | 0  (0%) | 0  (0%) | 0  (0%) | 0  (0%) |

Denominator of screening responses is total number of screens, rather than the entire sample. Denominator of any counseling is entire sample. Denominator of counseling with any screening or counseling with no screening is total number of any counseling. Denominator of counseling with positive screening, negative screening, or no response to screening is total number of counseling with any screening. To mitigate any possibility of deductive disclosure, any non-zero results less than 10 were designated as “< 10” with certain corresponding results within the same column designated as “> 10” where applicable; this was not done for documented counseling without screening given the low risk of deductive disclosure in this setting.

**Table 3.** Patient sociodemographic of cohort, excluding one outlier clinic site of 18 total, comparing those who responded to screens with those who did not respond to screens.

| **Characteristics** | | **No. (%)** | | |
| --- | --- | --- | --- | --- |
|  |  | Responded to screen with positive or negative response  (N=17,466) | Did not respond  (N=10,170) | p-value^b^ |
| **Patient sociodemographic characteristics** | **Female sex** | 10,061 (57.6%) | 5,579 (54.9%) | < 0.01 |
|  | **Age group, years** | | | |
|  | 18-44 | 5,447 (31.2%) | 2,943 (28.9%) | < 0.01 |
|  | 45-64 | 6,677 (38.2%) | 3,958 (38.9%) | 0.26 |
|  | ≥65 | 5,342 (30.6%) | 3,269 (32.1%) | 0.01 |
|  | **Race and ethnicity** | | | |
|  | Hispanic, any race | 475 (2.7%) | 355 (3.9%) | < 0.01 |
|  | Non-Hispanic, Asian | 1,359 (7.8%) | 982 (8.2%) | 0.27 |
|  | Non-Hispanic, Black | 957 (5.5%) | 764 (11.0%) | < 0.01 |
|  | Non-Hispanic, Other | 519 (3.0%) | 433 (3.6%) | < 0.01 |
|  | Non-Hispanic, White | 14,156 (81.0%) | 11,707 (73.4%) | < 0.01 |

^a^ This sample excludes one of 18 clinic sites given outlier result of near zero screening as shown in Table S2.

^b^ Two-proportion Z-tests were used to compare proportions between the two groups with corresponding p-values shown here.

**Figure 1.** Odd ratios of logistic regression model correlates of firearm safety counseling given positive screening, including all 18 clinics. Odds ratios are adjusted for all covariates in the model.
